# Editorial: Bio-engineered organs and grafts for clinical transplantation

**DOI:** 10.3389/frtra.2025.1763397

**Published:** 2026-01-09

**Authors:** Siba Haykal

**Affiliations:** Plastic & Reconstructive Surgery, Yale University, New Haven, CT, United States

**Keywords:** auto transplantation, bio engineering, eplet matching, *ex vivo* machine perfusion, tolerance induction

The first successful kidney transplant was performed in 1954 by Joseph Murray. His first donor and recipient patients were identical twins. The first kidney allograft was performed five years later, in 1959, using whole-body irradiation. However, the major leap in organ transplantation occurred in 1962, when azathioprine (the Prodrug of 6-mercaptopurine) made it possible for Murray to perform a kidney transplant from an unrelated diseased donor (1). These medical advancements led to Nobel Prizes, with Sir James W. Black, Gertrude B. Elion, and George H. Hitchings receiving the award in 1988 for their work in designing and developing purine analogues and their use in immunosuppression and cancer therapy (2). In 1990, Joseph Murray and E. Donnall Thomas were honoured for their pioneering work in organ and bone marrow transplantation (3). Since the 1950s, the field of transplantation has expanded to include livers, pancreas, lungs, hearts, and intestines. The latest advancement occurred in 1998, when the first successful hand transplantation opened the field of vascularized composite tissue allotransplantation (VCA) (4). Since then, the field has rapidly evolved in the first quarter of the 21st century to include face (5), larynx (6), uterus (7), urogenital and abdominal wall (6).

Although there have been significant improvements in surgical techniques and immunological understanding, key challenges remain, such as “shortage of donor organs”, “ischemia-reperfusion injuries”, and “lifelong immunosuppression”. Recent research has focused on addressing these issues. For example, the use of machine perfusion can reduce ischemia-reperfusion injury, increase donor organ availability, and potentially facilitate donor organ engineering and immune modification. Advanced surgical techniques now allow for the repair or treatment of failing organs, which can then be transplanted back autologously, reducing the reliance on donor organs. Additionally, immune tolerance induction methods and a deeper understanding of molecular structures and antigen-antibody interactions are leading to improved donor-recipient matching and better outcomes. In this research topic collection, we showcase four papers that outline the recent advancements in organ repair and transplantation across various contexts and aim to highlight how emerging technologies can improve graft quality and reduce the burden of immunosuppression so that more patients can safely benefit from transplantation.

Duru et al. reviewed the current state of *ex vivo* machine perfusion, which already has established clinical applications for thoracoabdominal organs. They discussed how these technologies are now being adapted for VCA grafts. Their scoping review summarizes reported perfusion models including extremities and flaps. By analyzing variables such as temperature, perfusate composition, circuit design, and outcome metrics, the authors provide a comprehensive overview of how different configurations influence muscle viability, vascular stability, edema control, and ultimately the feasibility of extended preservation times. Moreover, these platforms allow genetic constructs to be delivered to the graft during the preservation opening a broad spectrum of possibilities such as insertion of reporter genes fir early detection of rejection, or various genetic modulations to counteract ischemia reperfusion injury pathways or even correction of genetic disorders (8).

In the second paper of the collection, Sun et al. comprehensively outline the advancements of tolerance induction strategies from the VCA perspective. They first outline the key immunologic mechanisms that lead to rejection, then, as VCA contains different tissue types such as skin, muscle and bone, they outline the different immunological profile of these tissues. Strategies like mixed chimerism, T-cell depletion, regulatory T cells, vascularized bone marrow, and co-stimulation blockade are discussed. Most success so far has come from rodent models, while translation into large animals and non-human primates remains challenging due to stronger alloimmune responses and safety concerns with conditioning regimens.

In the next paper, Abulaiti et al. provide a comprehensive bibliometric and visualization analysis of digestive system autotransplantation, focusing on autologous liver, pancreatic, and small-intestine procedures in the last 20 years. These Autologous transplant approaches within the digestive system increasingly reflect a repair-based, bioengineering mindset rather than simple resection. In the liver, *ex vivo* resection with autotransplantation enables precise tumor removal and reconstruction of vasculature and biliary structures under controlled conditions, effectively “repairing” the organ before reimplantation. Similarly, total pancreatectomy with islet autotransplantation (TPIAT) embodies a functional tissue-sparing strategy: diseased tissue is removed, while endocrine function is preserved through autologous islet reinfusion. Autologous small intestine transplantation follows the same principle, allowing the reuse of native intestine to recover nutrient absorption in otherwise untreatable cases. Progress in perioperative care and tissue protection continues to push this highly complex procedure toward safer and more durable outcomes. Overall, autotransplantation is a promising approach for repairing or treating organs.

Finally, Stögner et al. provide a review of molecular-level HLA matching, reflecting the rapid shift from antigen-based typing toward eplet-based compatibility assessment in solid organ transplantation. Eplets have been recognized since the early 2000s as molecular structures are better recognized and studied. The authors looked at 98 studies that encompass nearly 300,000 transplant recipients across kidney, heart, lung, liver, and pancreas grafts confirms that class II eplet mismatch—particularly at HLA-DQ and HLA-DR—is a strong predictor of *de novo* donor-specific antibodies, antibody-mediated rejection, and graft dysfunction. Importantly, the review highlights why translation into policy remains slow: variability in mismatch algorithms, typing resolution, immunosuppressive practices, and organ-specific immunobiology lead to inconsistent thresholds and effect sizes. Their work reinforces that precision histocompatibility will be central to reducing rejection toxicity and improving long-term graft survival across all transplant types.

Together, this collection illustrates how the field of transplantation is rapidly evolving from organ replacement toward organ repair, regeneration, and immunologic precision*.* Continued convergence of bioengineering, immune modulation, and advanced preservation technologies will be essential to expand graft availability, reduce toxicity, and ultimately enable long-term graft health with minimal lifelong burden.

## References

[1] Nordham KD Ninokawa S. The history of organ transplantation. Proc (Bayl Univ Med Cent). (2022) 35(1):124–8. 10.1080/08998280.2021.1985889 34970061 PMC8682823

[2] Marx JL. The 1988 Nobel prize for physiology or medicine. Science. (1988) 242(4878):516–7. 10.1126/science.3051384 3051384

[3] Cash MP Dente CJ Feliciano DV. Joseph E. Murray (1919–): Nobel Laureate, 1990. Arch Surg. (2005) 140(3):270–2. 10.1001/archsurg.140.3.270 15781791

[4] Petruzzo P Lanzetta M Dubernard JM Margreiter R Schuind F Breidenbach W The international registry on hand and composite tissue transplantation. Transplantation. (2008) 86(4):487–92. 10.1097/TP.0b013e318181fce8 18724213

[5] Siemionow M. The past the present and the future of face transplantation. Curr Opin Organ Transplant. (2020) 25(6):568–75. 10.1097/MOT.0000000000000812 33044347

[6] Wu S Xu H Ravindra K Ildstad ST. Composite tissue allotransplantation: past, present and future-the history and expanding applications of CTA as a new frontier in transplantation. Transplant Proc. (2009) 41(2):463–5. 10.1016/j.transproceed.2009.01.027 19328904 PMC2858453

[7] Zitkute V Kvietkauskas M Leber B Strupas K Stiegler P Schemmer P. Ischemia and reperfusion injury in uterus transplantation: a comprehensive review. Transplant Rev. (2020) 34(3):100550. 10.1016/j.trre.2020.100550 32498979

[8] Filz Von Reiterdank I Bento R Hyun I Isasi R Wolf SM Coert JH Annual review of biomedical engineering designer organs: ethical genetic modifications in the era of machine perfusion. Annu Rev Biomed Eng. (2025) 27:101–28. 10.1146/annurev-bioeng-062824 39874605 PMC12229083

